# Reduced Interhemispheric Functional Connectivity in Obsessive–Compulsive Disorder Patients

**DOI:** 10.3389/fpsyt.2019.00418

**Published:** 2019-06-13

**Authors:** Ke Deng, Tianfu Qi, Jian Xu, Linlin Jiang, Fengrui Zhang, Nan Dai, Yuqi Cheng, Xiufeng Xu

**Affiliations:** ^1^Department of Psychiatry, First Affiliated Hospital of Kunming Medical University, Kunming, China; ^2^Department of Medical Imaging, First Affiliated Hospital of Kunming Medical University, Kunming, China; ^3^Department of Rheumatology, First Affiliated Hospital of Kunming Medical University, Kunming, China; ^4^Yunan Key Laboratory of Laboratory Medicine, Kunming, China

**Keywords:** obsessive–compulsive disorder (OCD), r-fMRI, functional connectivity (FC), interhemispheric functional connectivity, homotopic connectivity, voxel-mirrored homotopic connectivity (VMHC)

## Abstract

**Background:** Neuroimaging studies have shown that the high synchrony of spontaneous neural activity in the homotopic regions between hemispheres is an important functional structural feature of normal human brains, and this feature is abnormal in the patients with various mental disorders. However, little is known about this feature in obsessive–compulsive disorder (OCD). This study aimed to further analyze the underlying neural mechanisms of OCD and to explore whether clinical characteristics are correlated with the alerted homotopic connectivity in patients with OCD.

**Methods:** Using voxel-mirrored homotopic connectivity (VMHC) during resting state, we compared 46 OCD patients and 46 healthy controls (HCs) matched for age, gender, and education level. A partial correlation analysis was used to investigate the relationship between altered VMHC and clinical characteristics in patients with OCD.

**Results:** Patients with OCD showed lower VMHC than HCs in fusiform gyrus/inferior occipital gyrus, lingual gyrus, postcentral gyrus/precentral gyrus, putamen, and orbital frontal gyrus. A significant positive correlation was observed between altered VMHC in the angular gyrus/middle occipital gyrus and illness duration in patients.

**Conclusions:** Interhemispheric functional imbalance may be an essential aspect of the pathophysiological mechanism of OCD, which is reflected not only in the cortico-striato-thalamo-cortical (CSTC) loop but also elsewhere in the brain.

## Introduction

Resting-state functional magnetic resonance imaging (r-fMRI) technology indirectly reflects the intrinsic, spontaneous neural activity of the brain and can be used to measure resting-state functional connectivity (RSFC) between brain regions directly (1). Voxel-mirrored homotopic connectivity (VMHC) is an R-fMRI analysis method proposed by Zuo XN in recent years (2). VMHC quantifies the RSFC between each voxel in one hemisphere and its mirrored counterpart in the other hemisphere (i.e., homotopic RSFC). R-fMRI studies have discovered the high synchronicity of spontaneous activity between homotopic regions in healthy human brains, showing regional differences consistent with brain function levels (3, 4). Furthermore, a VMHC study using a large sample of healthy subjects (214 cases) demonstrated a robust homotopic RSFC architecture that exhibits regionally specific age- and sex-related changes across the lifespan (2). Therefore, high synchronicity of spontaneous neural activity between homotopic regions is considered an important feature of normal brain function.

Obsessive–compulsive disorder (OCD) is a common, typically chronic disorder marked by intrusive and disturbing thoughts (obsessions) and repetitive behaviors (compulsions) that the person feels driven to perform (5). The lifetime prevalence is about 1–3%. The patients understand that these compulsive symptoms are unreasonable, unnecessary, but they are unable to control or get rid of them, thus falling into anxiety and pain (6). Furthermore, OCD is characterized by intense emotional arousal and executive control impairments (7). These two mechanisms influence each other and are responsible for maintaining the obsessive–compulsive cycle (8). Although the exact pathophysiological mechanism of OCD is not fully understood, it is currently considered to be closely related to alterations in the cortico-striato-thalamo-cortical (CSTC) circuitry, which includes some main gray matter (GM) nodes such as the orbitofrontal cortex (OFC), dorsolateral prefrontal cortex (DLPFC), anterior cingulate cortex (ACC), striatum, and thalamus (9, 10). The majority of previous OCD r-fMRI studies tended to use seed-based FC analyses with a focus on local abnormalities, especially within the fronto-striatal circuit. Recently, studies using the VMHC method explored altered homotopic RSFC in a variety of mental illnesses, such as depression, schizophrenia, sleep disorder, dementia, addiction barrier, bipolar disorders, and phobia (11–17). However, little is known about changes in the homotopic RSFC in OCD. Some early studies have suggested that OCD patients may have interhemispheric structural and functional abnormalities. Two studies on interhemispheric structural connectivity of the OCD patients found that abnormal corpus callosum (CC) morphology and fractional anisotropy (FA) (18, 19). Both increased and decreased FA values in the CC were reported in a meta-analysis of Diffusion tensor imaging (DTI) studies on OCD (20), which suggested that changes in the microstructure of the CC may be involved in the process of obsessions and compulsions (21). It is noteworthy that a neuropsychological study of OCD found that microstructural damage was significantly associated with cognitive performance in intra-hemispheric bundles but not in CC (22). Some Electroencephalography (EEG) studies found that compared with healthy controls (HCs), OCD patients had abnormal electrical activity on one side of the hemisphere (23–25) (left hemisphere or right hemisphere). A study of transcranial magnetic stimulation (TMS) found that stimulation of the right DLPFC resulted in the relief of OCD symptoms, while stimulation of the left DLPFC did not resolve (26). Additionally, a Positron emission tomography (PET) study found that left and right hemisphere DLPFC showed opposite perfusion responses in acute symptomatic OCD patients (27). Evidence from neurosurgery indicated that symptomatic improvements were observed in patients with OCD after right anterior capsulotomy, but not after left anterior capsulotomy (28, 29). Nonetheless, the deficits in these patients seem not to be related to a specific lateralized dysfunction of a particular hemisphere, but probably due to a functional inter-hemisphere imbalance (30). Although all of the above findings suggested that there may be a special interhemispheric functional effect in OCD patients, there is almost no R-fMRI study that specifically clarify clarifies what is the interhemispheric functional connectivity pattern of OCD patients compared to healthy controls.

In this study, we used R-fMRI combined with the VMHC approach to explore changes in homotopic connectivity in OCD patients. We compared the VMHC differences between OCD patients and HCs, and between treated and drug-naive OCD patients. The aims of this study were to verify that OCD patients had significant VMHC abnormalities (31) and to examine whether medical treatment affects the altered VMHC in OCD. Moreover, we expected to explore a relationship between altered VMHC values and the clinical characteristics of OCD patients.

## Materials and Methods

### Participants

This study has been approved by the Ethics Committee of Kunming Medical University (ClinicalTrials.gov: NCT01298622). The researchers introduced all participants to the purpose, content, potential risks, and benefits of the study; the principle of voluntary participation; and the anonymity and confidentiality of the research. All participants signed informed consent.

A total of 49 OCD patients (including the outpatients and inpatients) were recruited from the First Affiliated Hospital of Kunming Medical University from October 2011 to December 2016. Inclusion criteria were as follows: a) comply with *Diagnostic and Statistical Manual of Mental Disorders—Fourth Edition* (DSM-IV) criteria for OCD based on the Structured Clinical Interview; b) Yale–Brown Obsessive–Compulsive Scale (Y-BOCS) total score ≥16 points, and Hamilton Depression Rating Scale (HAMD) score <18 points; c) age ranges from 18 to 60 years old; d) preference for using the right hand; e) all the OCD patients’ patients’ obsessive–compulsive symptoms were not caused by another mental disorder or physical disease; f) exclude organic brain diseases and major physical illnesses; g) no metal implants in the body. When performing MRI scans, 25 of them are first-episode untreated patients; 24 had received psychiatric medication for more than 4 weeks. The vast majority of the drugs taken by 24 patients are SSRI (selective serotonin reuptake inhibitors) drugs. Of the 24 patients, 9 patients took sertraline, 5 patients took multiple drugs (two kinds of SSRI and venlafaxine or two kinds of SSRI and clomipramine), 3 patients took sertraline and fluoxetine, 3 patients took paroxetine, 2 patients took sertraline and paroxetine, and 2 patients took fluoxetine.

We also enrolled 46 healthy controls from society during the period from September 2011 to 2017. Entry criteria were as follows: a) age 18 to 60 years old; b) right-handed; c) no mental illness meeting the diagnostic criteria; d) no family history of mental illness; e) gender, age, handedness, and education years are matched with the OCD group; f) no metal implants in the body.

The obsessive–compulsive symptoms, depressive symptoms, and anxiety symptoms of the OCD group and the HC group were evaluated using the Yale–Brown Obsessive Compulsive Scale (Y-BOCS), Hamilton Depression Rating Scale-17 items (HAMD-17), and Hamilton Anxiety Rating Scale (HAMA). The above evaluations were performed by two experienced clinical psychiatrists.

### MRI Acquisition

MRI images were obtained using a Philips Achieva 3.0-T MRI scanner in the First Affiliated Hospital of Kunming Medical University. The participants were required to remain motionless and awake with their eyes closed. Soft earplugs and foam pads were used to reduce scanner noise and head motion. A gradient-echo sequence was also used to obtain high-resolution T1-weighted structural MRI images with the following parameters: time of repetition (TR)/time of echoing (TE) = 2,500/80 ms, slice thickness = 6 mm, field of vision (FOV) = AP (250 mm) × Right/left (RL) (193 mm) × Foot/head (FH) (142 mm), matrix size = 128 × 128, flip angle = 90°, slices = 16, gap = 2 mm, scan duration time = 45 s. Normal T1-weighted MRI scans were first performed to exclude obvious structural abnormalities. The resting-state functional images were acquired by using an echo-planar imaging (EPI) sequence with the following parameters: TR/TE = 2,200/35 ms, flip angle = 90°, FOV = 230 × 230 mm, matrix = 128 × 128, slice thickness = 3.0 mm without interlayer spacing, slices = 50, scan duration time = 17 min 40 s.

### MRI Preprocessing

Functional magnetic resonance imaging (fMRI) data preprocessing were performed using the statistic parametric mapping software package (SPM12, http://www.fil.ion.ucl.ac.uk/spm) running in the Matlab 2012a (MathWorks, Natick, MA, USA) and in the Data Processing Assistant for Resting-State fMRI (DPARSF, http://rfmri.org/DPARSF) (32). The steps of preprocessing were as follows: a) format conversion: convert the Digital imaging and communications in medicine (DICOM) format of the original image data into Neuroimaging informatics technology initiative (NIFTI) format; b) removal of the first 10 time points; c) time correction; d) head motion correction, data removal of average head motion translation >2 mm and/or rotation >2° (excluding two untreated OCD subjects and one drug-treated OCD subject); e) linearly register each subject’s T1 image to the corresponding functional image and then divide it into gray matter, white matter, and cerebrospinal fluid; f) removal of the influence due to covariates (24-head movement parameters, white matter signal, cerebrospinal fluid signal); g) Each of the abovementioned registered images was non-linearly registered to the MNI (Montreal Neurological Institute) standard space and resampled to a voxel size of 3 × 3 × 3 mm^3^; h) the signal was linearly detrended and bandpass filtered at 0.01–0.08 Hz to reduce low-frequency drifts and high-frequency physiological noise (i.e., respiratory and cardiac) (33).

### VMHC Calculation

Before using the Data processing & analysis for (resting-state) brain imaging (DPABI) software to calculate VMHC, a brain symmetry template was initially created to minimize the influence of geometric differences between the hemispheres on VMHC. Specifically, first, all 46 normalized T1 images of the healthy controls are averaged to create an average normalized T1 image; then, this average T1 image is re-averaged using its left and right mirrored versions to generate a particular group symmetric template. Then, this group symmetric template is applied to the 46 standardized images after the above pre-processing steps and then smoothed by a Gaussian kernel of 4-mm full width and half maximum (FWHM). VMHC is then calculated to obtain VMHC maps and zVMHC maps (Fisher *z*-transformation) for each subject. For each subject, VMHC was computed as Pearson correlation coefficient between each voxel’s residual time series and that of a corresponding voxel in the opposite hemisphere as described in a previous study. Similarly, the OCD group was processed to obtain a group symmetric template and 46 zVMHC maps. More details about the VMHC method were given in the article (2).

### Statistical Analysis

Based on the statistical module in the DPABI software, group differences on zVMHC maps between the patients and the controls were calculated by using two-sample *t* tests, after adjustment for age, gender, education, mean framewise displacement (mean FD), and medication status. Given that a prior study has suggested that RSFC could be affected by micromovements from volume to volume (34), we calculated the mean framewise displacement (FD) values for each subject, which can reflect the temporal derivative of the movement parameters. FD values were calculated for each item as described in a previous study (34). The threshold for significance was set at *p* < 0.005 (two-tailed) and 5,000 iterations corrected by the TFCE + PT (Permutation test with Threshold-Free Cluster Enhancement) methods in the PALM tool (PALM—Permutation Analysis of Linear Models) (35, 36). Then, we got a corrected T-map. To observe the clinical relevancies of VMHC, the voxel-wise Pearson correlation analysis was calculated between each patient’s zVMHC map and clinical characteristics (Y-BOCS total score, Y-BOCS obsession score, Y-BOCS compulsion score, and illness duration) by using the abovementioned corrected T-map as a mask. Age, gender, mean FD, HAMD score, and HAMA score were applied as covariates of no interest. The threshold for significance was also set at *p* < 0.005 (two-tailed) and 5,000 iterations corrected by the TFCE + PT methods. Then, we extracted the mean zVMHC values of the brain regions exhibiting significant correlations between abnormal VMHC and clinical characteristics to get the scatter plot. Considering that SSRI may affect VMHC, two-sample *t* tests were used to compare differences in zVMHC maps between 23 treated and 23 drug-naïve OCD patients, controlling for age, gender, education, and mean FD. The threshold for significance was corrected for TFCE + PT at *p* < 0.05 (two-tailed).

## Results

### Demographics and Clinical Characteristics

The data of three patients (two untreated OCD subjects and one drug-treated OCD subject) were excluded from the analyses due to excessive head movement. Hence, the final samples included 46 patients (23 untreated OCD subjects and 23 drug-treated OCD subjects) and 46 controls. There were no statistical differences in gender, age, education level, and mean FD between 46 OCD and 46 HCs (see **Table 1**). Similarly, there were no statistical differences in gender, age, education level, obsessive–compulsive symptoms, depressive symptoms, anxiety symptoms, and mean FD between two patient groups (see **Table 1**).

**Table 1:** Demographic and clinical characteristics of participants.

Demographic data	OCD patients (46)		HCs (46)	t/χ² value	p value
**Age (years)**	30.39 ± 10.68		31.83 ± 10.27	−0.657	0.513^b^
**Gender (male/female)**	26/20		26/20	0.000	1.000^a^
**Education (years)**	12.70 ± 2.97		13.83 ± 3.47	−1.679	0.097^b^
**Illness duration (months)**	49.83 ± 51.54		NA	NA	NA
**Y-BOCS total score**	28.85 ± 6.56		10.00 ± 0.00	19.490	<0.001^b^
**Y-BOCS obsession score**	15.17 ± 3.83		5.00 ± 0.00	18.037	<0.001^b^
**Y-BOCS compulsion score**	13.89 ± 4.88		5.00 ± 0.00	12.364	<0.001^b^
**HAMD score**	10.20 ± 4.87		0.52 ± 0.78	13.315	<0.001^b^
**HAMA score**	10.35 ± 4.67		0.65 ± 0.71	13.916	<0.001^b^
**Mean FD**	0.096 ± 0.031		0.088 ± 0.025	1.262	0.210^b^
	**Unmedicated OCD (23)**	**Medicated OCD (23)**			
**Age (years)**	27.83 ± 10.53	32.96 ± 10.43		−1.660	0.104^b^
**Gender (male/female)**	11/12	15/8		1.415	0.234^a^
**Education (years)**	13.09 ± 3.09	12.30 ± 2.87		0.891	0.378^b^
**Illness duration (months)**	49.39 ± 60.18	50.26 ± 42.55		−0.057	0.955^b^
**Y-BOCS total score**	27.61 ± 6.16	30.09 ± 6.84		−1.291	0.204^b^
**Y-BOCS obsession score**	15.22 ± 4.12	15.13 ± 3.60		0.076	0.940^b^
**Y-BOCS compulsion score**	12.83 ± 4.74	14.96 ± 4.88		−1.502	0.140^b^
**HAMD score**	10.43 ± 5.00	9.96 ± 4.83		0.330	0.743^b^
**HAMA score**	10.00 ± 3.92	10.70 ± 5.39		−0.501	0.619^b^
**Mean FD**	0.093 ± 0.035	0.098 ± 0.028		−0.476	0.636^b^

Y-BOCS, Yale–Brown Obsessive–Compulsive Scale; HAMD, Hamilton Depression Rating Scale; HAMA, Hamilton Anxiety Scale; Mean FD, mean frame-wise displacement; HC, healthy controls; OCD, obsessive–compulsive disorder; NA, not available. ^a^The p value for gender distribution was obtained by chi-square test. ^b^The p values were obtained by two-sample t tests.

### VMHC Differences Between Groups

**Figure 1:**
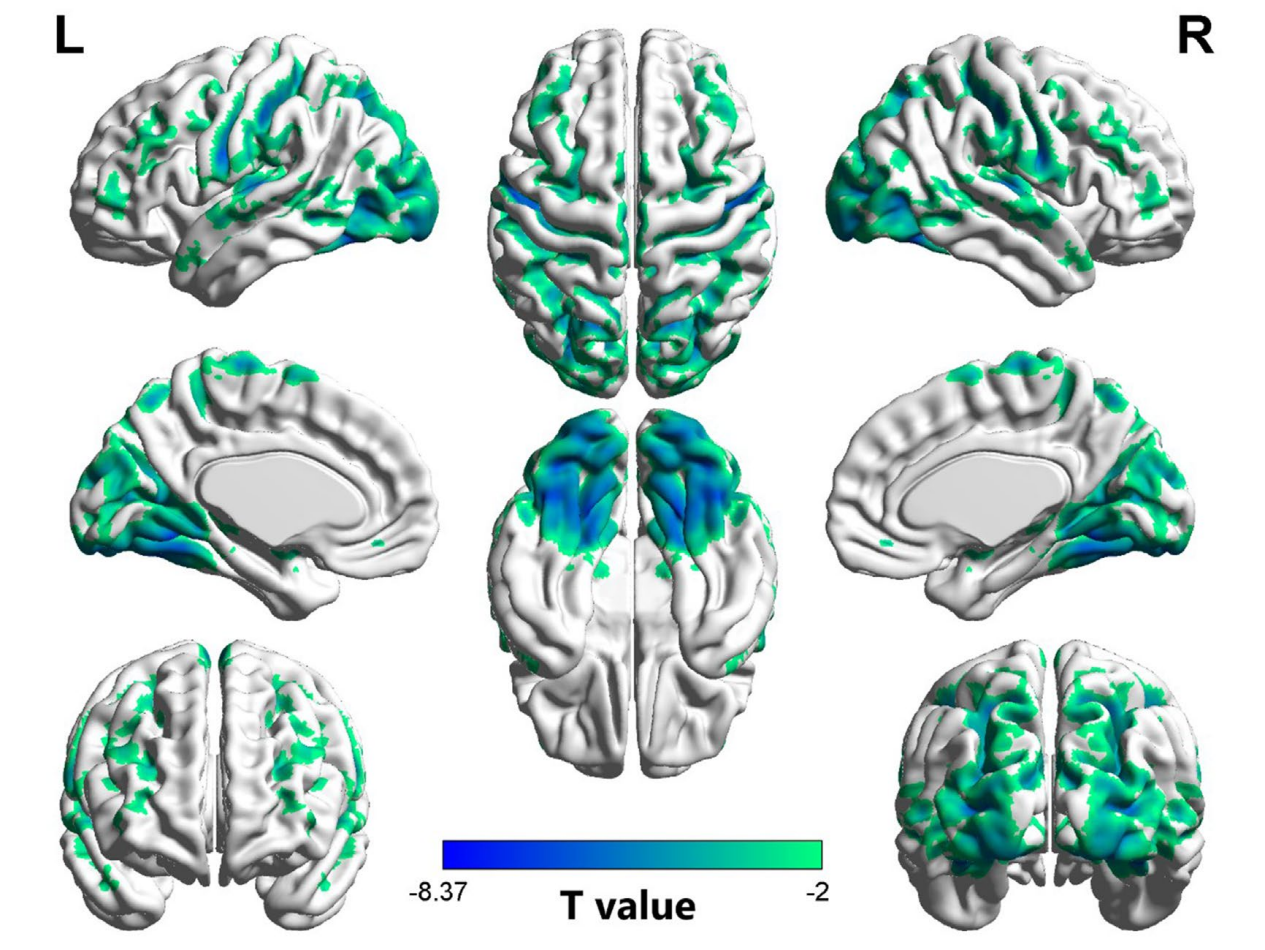
Regions with decreased homotopic connectivity in obsessive–compulsive disorder (OCD) patients compared to healthy controls. L: left; R: right.

As shown in **Table 2** and **Figure 1**, compared to the controls, the OCD patients showed significantly decreased VMHC in the fusiform gyrus/inferior occipital gyrus (*t* = −8.371, *p* < 0.005), lingual gyrus (*t* = −7.653, *p* < 0.005), postcentral gyrus/precentral gyrus (*t* = −7.701, *p* < 0.005), putamen (*t* = 4.321, *p* < 0.005), and orbital frontal gyrus (OFC) (*t* = 4.617, *p* < 0.005). No regions showed increased VMHC in the patients relative to controls. Moreover, there were no significant differences in VMHC when comparing the medicated and unmedicated patient sub-groups.

**Table 2:** Regions showing significant differences in VMHC between OCD patients and HCs.

Region	BA	Peak MNI coordinates (*x*, *y*, *z*)	*t* value	Cluster size (voxel)
**Fusiform Gyrus/Inferior Occipital Gyrus**	19/37	± 39 −63 −15	−8.371	529
**Postcentral Gyrus/Precentral Gyrus**	3	± 57 −9 33	−7.701	721
**Lingual Gyrus**	37	± 22 −54 −11	−7.653	445
**Putamen**	NA	± 15 12 −3	−4.321	146
**Orbital Frontal Gyrus**	11	± 9 42 −12	−4.617	50

VMHC, voxel-mirrored homotopic connectivity; BA, Brodmann area; MNI, Montreal Neurological Institute; NA, not available. Comparisons are adjusted for age, sex, education, and mean FD.

### Correlation Between Altered VMHC and Clinical Characteristics

**Figure 2:**
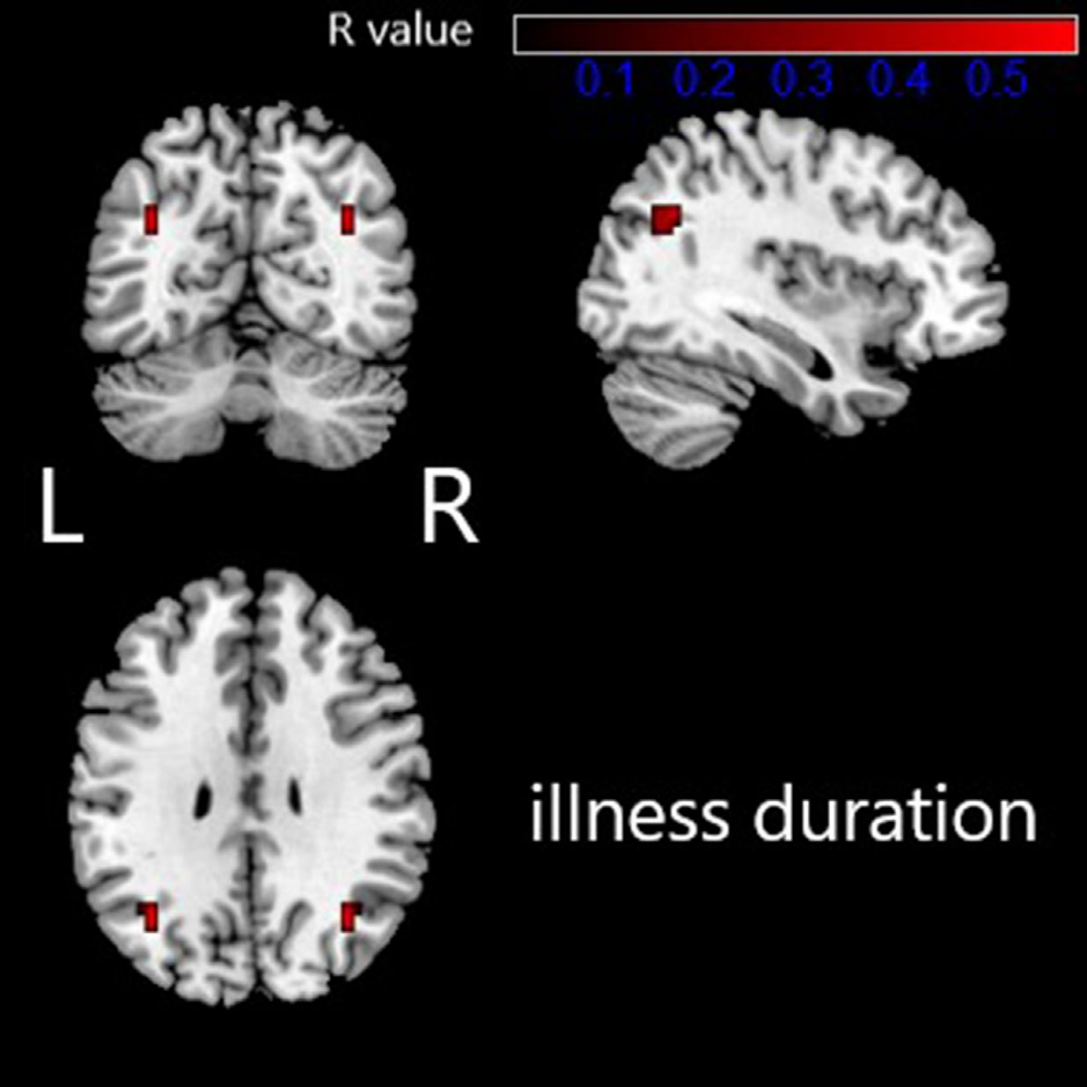
Regions exhibiting significantly positive correlations between VMHC value and illness duration in OCD patients are presented as color overlays. The color bar represents *R* values. L: left; R: right.

**Figure 3:**
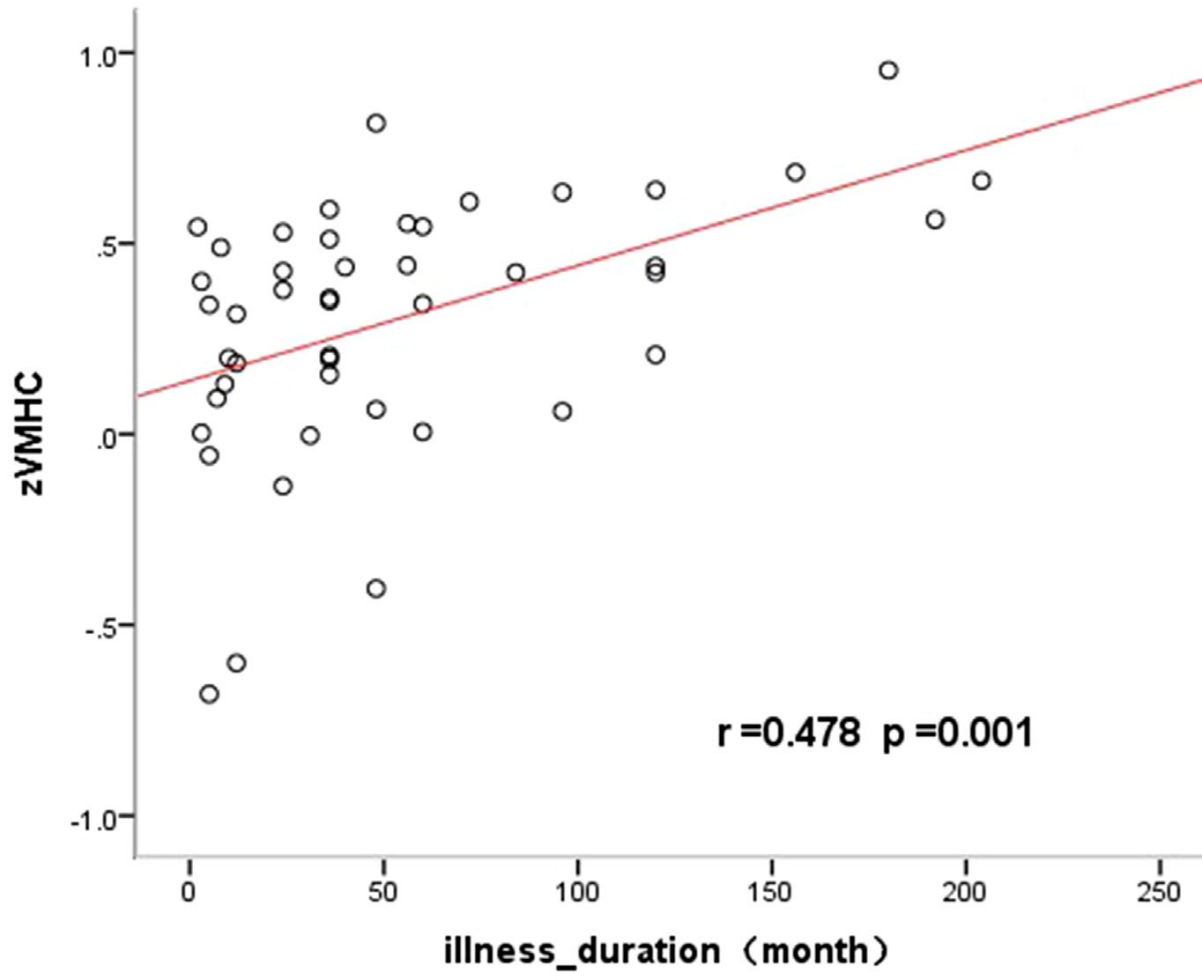
Significantly positive correlations between the VMHC values and the illness duration in the angular gyrus/middle occipital gyrus in OCD.

The altered VMHC in the angular gyrus/middle occipital gyrus was found to be significantly positively correlated with disease duration (*R* = 0.568, *p* < 0.05, see **Table 3** and **Figures 2** and **3**). No other brain regions were found to have a significant correlation between VMHC values and symptom severity (Y-BOCS total score, Y-BOCS obsession score, and Y-BOCS compulsion score).

**Table 3:** Regions showing significant correlations between VMHC value and illness duration in OCD patients.

Region	BA	Peak MNI coordinates (*x*, *y*, *z*)	*R* value	Cluster size (voxel)
**Angular Gyrus/Middle Occipital Gyrus**	39	± 36 −63 30	0.568	9

## Discussion

In this study, we found decreased VMHC within CSTC circuitry (putamen and OFC), the fusiform gyrus/inferior occipital gyrus, lingual gyrus, and postcentral gyrus/precentral gyrus in patients with OCD relative to controls. The altered VMHC was not correlated with the clinical severity of OCD symptoms in the patient group but had a significant positive correlation with disease duration. However, no brain regions showed significant differences in VMHC between the SSRI-treated and drug-naive patients.

Similar to this study, Wang et al. reported that patients with OCD had a lower VMHC in the CSTC circuitry (thalamus and OFC) than HCs, but no abnormal VMHC was found to be associated with the severity of clinical symptoms (after correction), nor was there a difference in VMHC between the SSRI-treated and drug-naive patients (37). However, inconsistent with this study, VMHC abnormalities in the fusiform gyrus/inferior occipital gyrus, lingual gyrus, and postcentral gyrus/precentral gyrus in OCD patients were not reported by Wang et al., which may be due to sample heterogeneity and analytical methods. For example, in this study, we calculated the group differences in VMHC between OCD patients and HCs based on whole brain voxels, while Wang et al. was based on the voxels that showed significant VMHC in any of the two groups (OCD patients and HCs) (37). To sum up, we found that OCD patients had significantly weaker homotopic RSFC than healthy controls, which is consistent with the findings in other various mental illnesses (11, 12, 14–17), meaning that homotopic RSFC abnormalities may be as critical pathophysiological features of mental illness as Rest state network (RSN) abnormalities are (38–41).

Although recent neuroimaging studies emphasize the abnormal structures and functions of the CSTC circuitry in OCD, these previous studies have not explored the VMHC changes in the CSTC circuitry. Therefore, the VMHC alterations in the striatum and OFC reported in this study may provide new evidence for abnormalities in the CSTC circuitry in OCD. Recent studies have been identifying additional brain correlates associated with OCD symptomatology outside of CSTC circuitry (42), with these findings contributing to the generation of new hypotheses for the OCD pathogenesis (31, 43). Therefore, the VMHC alterations outside the CSTC loop found in this study seem to confirm that the pathophysiological mechanism of OCD may be not only related to the CSTC loop. A recent meta-analysis reported orbitofrontal and striatal dysfunction during executive control in OCD patients (44), as well as abnormalities in activation within precentral/postcentral and occipital lobe regions. In fact, these regions are also implicated in OCD during other tasks such as reward tasks (45, 46), psychomotor vigilance tasks (47), and emotional processing tasks (48–50). Therefore, based on the reduction of VMHC in the OFC, striatum, postcentral/precentral gyrus, and IOG/fusiform gyrus found in our study, we speculate that the abnormal VMHC may be related to cognitive/executive functional deficits and emotional processing impairment in OCD patients, an idea that should be tested directly in future research.

The CC is the main commissural fiber bundle mediating interhemispheric transfer (51), and broad reductions of homotopic connectivity after dissection of the CC (52) underscore the relevance of this structure for interhemispheric transfer. The CC has therefore been identified as an important structural basis of interhemispheric RSFC. A further supportive finding in the reviewed papers on OCD is the substantiation of microstructural abnormalities in the CC with strong evidence for increased and decreased FA (20, 53). Similarly, the results of our previous DTI studies based on the same OCD patients also support altered FA in the CC in OCD patients compared with HCs. Furthermore, the moderate correlations between VMHC and FA of the CC have been reported in patients with migraine and multiple sclerosis (54, 55). Therefore, these may suggest that reduced VMHC in OCD patients is based on obvious microstructural alterations of the CC, which should be further verified by implementing a correlation analysis between the altered VMHC and FA of the CC in OCD patients in future research.

To explore the effect of SSRI on the homotopic connectivity, we compared the group difference in VMHC between SSRI-treated and drug-naive OCD patients and found no differences in VMHC in any brain region. The findings were consistent with a recent similar study (37); these results might imply the limited effect of medication on regulating abnormal VMHC in OCD. However, as this is a cross-section study, further prospect study comparing the same group of patients before and after treatment is thus necessary to elucidate the exact effect of medication on VMHC in OCD patients.

The decreased VMHC in the angular gyrus/middle occipital gyrus was found to be positively correlated with the illness duration. This may be due to functional compensation during disease development. In fact, there is a lot of evidence that the duration of the disease can cause significant changes in brain structure and function in OCD patients. For example, illness duration has been found to be correlated with both hippocampus and left amygdala volume abnormalities in OCD (56). Furthermore, decreased left caudate nucleus–thalamus connectivity within the CSTC circuitry have been found to be positively correlated with the illness duration of OCD (57). Reduced connectivity in an emotion processing network spanning the left cerebellar lobule VI and the lingual gyrus has been reported to be correlated with illness duration (58). Changes in both the Regional Homogeneity (ReHo) within the OFC and the functional connectivity between the OFC and angular gyrus has been reported to be correlated negatively with OCD duration (59). However, since the results of the correlation analysis after multiple comparison correction showed that the cluster (9 voxels) was very small, the results from the present study should be interpreted with caution.

Up to now, this study is the second study to explore interhemispheric functional connectivity in OCD patients by using the VMHC method. The present study illustrates the interhemispheric functional imbalance in OCD patients, which should improve the understanding of OCD. In addition, the currently recommended method of TFCE + PT was used for multiple comparison corrections, which has been shown to control the false-positive rate to within 5% and to lead to the highest reproducibility when compared with other common thresholding methods (35).

## Limitations

Some limitations should be taken into consideration. Firstly, the relationship between altered VMHC and FA of the CC was not assessed in the present study. Future studies using a multimodal imaging method, such as voxel-based morphometry (VBM) and DTI, would help identify the unknown structural basis for VMHC alterations. Secondly, neuropsychological data, especially cognitive and behavioral information, were not collected in our study. The relationship between deficits in VMHC and cognitive dysfunction should be investigated in future research. Thirdly, the VMHC results in our study were obtained during resting state, and therefore, a task-oriented functional MRI study could provide a complementary view. Fourthly, although a rough assessment in the study did not reveal a significant effect of drug therapy on VMHC, longitudinal studies may be needed to clarify the effect of the drug on VMHC. Finally, a symmetrical standard template was applied with smoothed imaging data to improve the functional correlations between mirrored regions in the study. In general, the human brain is not symmetrical. Although morphometric asymmetry could not account for the reduced VMHC (15), the effects of methodological symmetry should not be overlooked.

## Conclusion

Interhemispheric functional imbalance, especially the imbalance in the CSTC circuit, is an essential aspect of the pathophysiological mechanism of OCD. Our results not only confirm that the CSTC circuit plays an important role in OCD, but also find that abnormal VMHC in areas other than the CSTC circuit is also involved in the pathophysiological mechanism of OCD.

## Data Availability Statement

All data sets generated for this study are included in the manuscript and supplemental documents.

## Ethics Statement

This study was carried out in accordance with the recommendations of the clinical trial guidelines of the Institutional Review Board of Kunming Medical University with written informed consent from all subjects. All subjects gave written informed consent in accordance with the Declaration of Helsinki. The protocol was approved by the Institutional Review Board of Kunming Medical University.

## Author Contributions

KD analyzed the data and wrote the draft. TQ and FZ collected the imaging data. JX and ND helped to revise the draft and polished it. LJ collected clinical data. YC and XX gave guidance and helped edit the manuscript. All authors read and approved the final manuscript.

## Funding

1. Founding of Yunnan Provincial Health Science and Technology Plan (2016NS026). 2. Yunnan Applied Basic Research Projects–Union Foundation [2017FE467(-167)]. 3. Innovative Research Team of Kunming Medical University (CXTD201705). 4. Middle and Young Aged Academic and Technology Leaders Reserve Personnel Foundation of Yunnan Province (2017HB062).

## Conflict of Interest Statement

The authors declare that the research was conducted in the absence of any commercial or financial relationships that could be construed as a potential conflict of interest.

## References

[1] Takamura T Hanakawa T Clinical utility of resting-state functional connectivity magnetic resonance imaging for mood and cognitive disorders. J Neural Transm (2017) 124(7):821–39. 10.1007/s00702-017-1710-2 28337552

[2] Zuo XN Kelly C Di Martino A Mennes M Margulies DS Bangaru S Growing together and growing apart: regional and sex differences in the lifespan developmental trajectories of functional homotopy. J Neuroscience (2010) 30(45):15034–43. 10.1523/JNEUROSCI.2612-10.2010 PMC2997358 21068309

[3] Salvador R Martinez A Pomarol-Clotet E Gomar J Vila F Sarro S A simple view of the brain through a frequency-specific functional connectivity measure. NeuroImage (2008) 39(1):279–89. 10.1016/j.neuroimage.2007.08.018 17919927

[4] Stark DE Margulies DS Shehzad ZE Reiss P Kelly AM Uddin LQ Regional variation in interhemispheric coordination of intrinsic hemodynamic fluctuations. J Neuroscience (2008) 28(51):13754–64. 10.1523/JNEUROSCI.4544-08.2008 PMC4113425 19091966

[5] Goodman WK Grice DE Lapidus KA Coffey BJ Obsessive–compulsive disorder. Psychiatr Clin North Am (2014) 37(3):257–67. 10.1016/j.psc.2014.06.004 25150561

[6] Ruscio AM Stein DJ Chiu WT Kessler RC The epidemiology of obsessive–compulsive disorder in the National Comorbidity Survey Replication. Mol Psychiatry (2010) 15(1):53–63. 10.1038/mp.2008.94 18725912 PMC2797569

[7] Abramowitz JS Jacoby RJJCPS Practice. Obsessive–compulsive disorder in the DSM-5. Clin Psychol-Sci Pr (2014) 21 (3):221–35. 10.1111/cpsp.12076

[8] Goncalves OF Batistuzzo MC Sato JR Real-time functional magnetic resonance imaging in obsessive–compulsive disorder. Neuropsychiatr Dis Treat (2017) 13:1825–34. 10.2147/NDT.S121139 PMC5513821 28744133

[9] Del Casale A Kotzalidis GD Rapinesi C Serata D Ambrosi E Simonetti A Functional neuroimaging in obsessive–compulsive disorder. Neuropsychobiology (2011) 64(2):61–85. 10.1159/000325223 21701225

[10] Menzies L Chamberlain SR Laird AR Thelen SM Sahakian BJ Bullmore ET Integrating evidence from neuroimaging and neuropsychological studies of obsessive–compulsive disorder: the orbitofronto-striatal model revisited. Neurosci Biobehav Rev (2008) 32(3):525–49. 10.1016/j.neubiorev.2007.09.005 PMC2889493 18061263

[11] Hermesdorf M Sundermann B Feder S Schwindt W Minnerup J Arolt V Major depressive disorder: findings of reduced homotopic connectivity and investigation of underlying structural mechanisms. Hum Brain Mapp (2016) 37(3):1209–17. 10.1002/hbm.23097 PMC6867499 26704348

[12] Agcaoglu O Miller R Damaraju E Rashid B Bustillo J Cetin MS Decreased hemispheric connectivity and decreased intra- and inter-hemisphere asymmetry of resting state functional network connectivity in schizophrenia. Brain Imaging Behav (2018) 12(3):615–30. 10.1007/s11682-017-9718-7 PMC5651208 28434159

[13] Zhou F Zhao Y Huang M Zeng X Wang B Gong H Disrupted interhemispheric functional connectivity in chronic insomnia disorder: a resting-state fMRI study. Neuropsychiatr Dis Treat (2018) 14:1229–40. 10.2147/NDT.S162325 PMC5957476 29795981

[14] Wang Z Wang J Zhang H McHugh R Sun X Li K Interhemispheric functional and structural disconnection in Alzheimer’s disease: a combined resting-state fMRI and DTI study. PloS One (2015) 10(5):e0126310. 10.1371/journal.pone.0126310 25938561 PMC4418835

[15] Kelly C Zuo XN Gotimer K Cox CL Lynch L Brock D Reduced interhemispheric resting state functional connectivity in cocaine addiction. Biol Psychiatry (2011) 69(7):684–92. 10.1016/j.biopsych.2010.11.022 PMC3056937 21251646

[16] Wang Y Zhong S Jia Y Zhou Z Wang B Pan J Interhemispheric resting state functional connectivity abnormalities in unipolar depression and bipolar depression. Bipolar Disord (2015) 17(5):486–95. 10.1111/bdi.12315 26241359

[17] Lai CH Wu YT The alterations in inter-hemispheric functional coordination of patients with panic disorder: the findings in the posterior sub-network of default mode network. J Affective Disord (2014) 166:279–84. 10.1016/j.jad.2014.05.022 25012442

[18] Ameis SH Lerch JP Taylor MJ Lee W Viviano JD Pipitone J A diffusion tensor imaging study in children with ADHD, autism spectrum disorder, OCD, and matched controls: distinct and non-distinct white matter disruption and dimensional brain–behavior relationships. Am J Psychiatry (2016) 173(12):1213–22. 10.1176/appi.ajp.2016.15111435 27363509

[19] Park HY Park JS Kim SH Jang JH Jung WH Choi JS Midsagittal structural differences and sexual dimorphism of the corpus callosum in obsessive–compulsive disorder. Psychiatry Res (2011) 192(3):147–53. 10.1016/j.pscychresns.2010.12.003 21543190

[20] Eng GK Sim K Chen SH Meta-analytic investigations of structural grey matter, executive domain-related functional activations, and white matter diffusivity in obsessive compulsive disorder: an integrative review. Neurosci Biobehav Rev (2015) 52:233–57. 10.1016/j.neubiorev.2015.03.002 25766413

[21] Jose D Narayanaswamy JC Agarwal SM Kalmady SV Venkatasubramanian G Reddy YC Corpus callosum abnormalities in medication-naive adult patients with obsessive compulsive disorder. Psychiatry Res (2015) 231(3):341–5. 10.1016/j.pscychresns.2015.01.019 25686521

[22] Spalletta G Piras F Fagioli S Caltagirone C Piras FJB, behavior. Brain microstructural changes and cognitive correlates in patients with pure obsessive compulsive disorder. Brain Behav (2014) 4(2):261–77. 10.1002/brb3.212 PMC3967541 24683518

[23] Kuskowski MA Malone SM Kim SW Dysken MW Okaya AJ Christensen KJ Quantitative EEG in obsessive–compulsive disorder. Biol Psychiatry (1993) 33(6):423–30. 10.1016/0006-3223(93)90170-I 8490069

[24] Flor-Henry P Yeudall LT Koles ZJ Howarth BG Neuropsychological and power spectral EEG investigations of the obsessive–compulsive syndrome. Biol Psychiatry (1979) 14(1):119–30. 420895

[25] Perros P Young ES Ritson JJ Price GW Mann P Power spectral EEG analysis and EEG variability in obsessive–compulsive disorder. Brain Topogr (1992) 4(3):187–92. 10.1007/BF01131149 1633056

[26] Greenberg BD George MS Martin JD Benjamin J Schlaepfer TE Altemus M Effect of prefrontal repetitive transcranial magnetic stimulation in obsessive–compulsive disorder: a preliminary study. Am J Psychiatry (1997) 154(6):867–9. 10.1176/ajp.154.6.867 9167520

[27] Rauch SL Jenike MA Alpert NM Baer L Breiter HC Savage CR Regional cerebral blood flow measured during symptom provocation in obsessive–compulsive disorder using oxygen 15-labeled carbon dioxide and positron emission tomography. Arch Gen Psychiatry (1994) 51(1):62–70. 10.1001/archpsyc.1994.03950010062008 8279930

[28] Lippitz BE Mindus P Meyerson BA Kihlstrom L Lindquist C Lesion topography and outcome after thermocapsulotomy or gamma knife capsulotomy for obsessive–compulsive disorder: relevance of the right hemisphere. Neurosurgery (1999) 44(3):452–8; discussion 8-60. 10.1097/00006123-199903000-00005 10069581

[29] Lippitz B Mindus P Meyerson BA Kihlstrom L Lindquist C Obsessive compulsive disorder and the right hemisphere: topographic analysis of lesions after anterior capsulotomy performed with thermocoagulation. Acta Neurochir Suppl (1997) 68:61–3. 10.1007/978-3-7091-6513-3_11 9233415

[30] Mataix-Cols D Junque C Vallejo J Sanchez-Turet M Verger K Barrios M Hemispheric functional imbalance in a sub-clinical obsessive–compulsive sample assessed by the Continuous Performance Test, Identical Pairs version. Psychiatry Res (1997) 72(2):115–26. 10.1016/S0165-1781(97)00074-7 9335202

[31] Goncalves OF Carvalho S Leite J Pocinho F Relvas J Fregni F Obsessive compulsive disorder as a functional interhemispheric imbalance at the thalamic level. Med Hypotheses (2011) 77(3):445–7. 10.1016/j.mehy.2011.06.004 21737205

[32] Chao-Gan Y Yu-Feng Z DPARSF: a MATLAB toolbox for “pipeline” data analysis of resting-state fMRI. Front Syst Neurosci (2010) 4:13. 10.3389/fnsys.2010.00013 20577591 PMC2889691

[33] Biswal B Yetkin FZ Haughton VM Hyde JS Functional connectivity in the motor cortex of resting human brain using echo-planar MRI. Magn Reson Med Sci (1995) 34(4):537–41. 10.1002/mrm.1910340409 8524021

[34] Power JD Barnes KA Snyder AZ Schlaggar BL Petersen SE Spurious but systematic correlations in functional connectivity MRI networks arise from subject motion. NeuroImage (2012) 59(3):2142–54. 10.1016/j.neuroimage.2011.10.018 PMC3254728 22019881

[35] Chen X Lu B Yan CG Reproducibility of R-fMRI metrics on the impact of different strategies for multiple comparison correction and sample sizes. Hum Brain Mapp (2018) 39(1):300–18. 10.1002/hbm.23843 PMC6866539 29024299

[36] Winkler AM Ridgway GR Douaud G Nichols TE Smith SM Faster permutation inference in brain imaging. NeuroImage (2016) 141:502–16. 10.1016/j.neuroimage.2016.05.068 PMC5035139 27288322

[37] Hua Wang Y Hui Chen Y Fang Li S Lv D Meng Zhao A Meng X Reduced Interhemispheric Resting-State Functional Homotopy in Obsessive-Compulsive Disorder Neuropsychiatry (2018) 8(3):1038–45. 10.4172/Neuropsychiatry.1000431

[38] Lowe MJ Dzemidzic M Lurito JT Mathews VP Phillips MD Correlations in low-frequency BOLD fluctuations reflect cortico-cortical connections. NeuroImage (2000) 12(5):582–7. 10.1006/nimg.2000.0654 11034865

[39] Lee MH Smyser CD Shimony JS Resting-state fMRI: a review of methods and clinical applications. AJNR Am J Neuroradiol (2013) 34(10):1866–72. 10.3174/ajnr.A3263 PMC4035703 22936095

[40] Wu QZ Li DM Kuang WH Zhang TJ Lui S Huang XQ Abnormal regional spontaneous neural activity in treatment-refractory depression revealed by resting-state fMRI. Hum Brain Mapp (2011) 32(8):1290–9. 10.1002/hbm.21108 PMC6870367 20665717

[41] Broyd SJ Demanuele C Debener S Helps SK James CJ Sonuga-Barke EJ Default-mode brain dysfunction in mental disorders: a systematic review. Neurosci Biobehav Rev (2009) 33(3):279–96. 10.1016/j.neubiorev.2008.09.002 18824195

[42] de Wit SJ Alonso P Schweren L Mataix-Cols D Lochner C Menchon JM Multicenter voxel-based morphometry mega-analysis of structural brain scans in obsessive–compulsive disorder. Am J Psychiatry (2014) 171(3):340–9. 10.1176/appi.ajp.2013.13040574 24220667

[43] Goncalves OF Marques TR Lori NF Sampaio A Branco MC Obsessive–compulsive disorder as a visual processing impairment. Med Hypotheses (2010) 74(1):107–9. 10.1016/j.mehy.2009.07.048 19695786

[44] Norman LJ Taylor SF Liu Y Radua J Chye Y De Wit SJ Error processing and inhibitory control in obsessive–compulsive disorder: a meta-analysis using statistical parametric maps. Biol Psychiatry (2018) 85(9):713–25. 10.1016/j.biopsych.2018.11.010 PMC6474799 30595231

[45] Norman LJ Carlisi CO Christakou A Murphy CM Chantiluke K Giampietro V Frontostriatal dysfunction during decision making in attention-deficit/hyperactivity disorder and obsessive–compulsive disorder. Biol Psychiatry Cognit Neurosci Neuroimaging (2018) 3(8):694–703. 10.1016/j.bpsc.2018.03.009 29706587 PMC6278892

[46] Marsh R Tau GZ Wang Z Huo Y Liu G Hao X Reward-based spatial learning in unmedicated adults with obsessive–compulsive disorder. Am J Psychiatry (2015) 172(4):383–92. 10.1176/appi.ajp.2014.13121700 PMC4382407 25526598

[47] Carlisi CO Norman L Murphy CM Christakou A Chantiluke K Giampietro V Disorder-specific and shared brain abnormalities during vigilance in autism and obsessive–compulsive disorder. Biol Psychiatry Cognit Neurosci Neuroimaging (2017) 2(8):644–54. 10.1016/j.bpsc.2016.12.005 PMC5685008 29167833

[48] Thorsen AL Hagland P Radua J Mataix-Cols D Kvale G Hansen B Emotional processing in obsessive–compulsive disorder: a systematic review and meta-analysis of 25 functional neuroimaging studies. Biol Psychiatry Cognit Neurosci Neuroimaging (2018) 3(6):563–71. 10.1016/j.bpsc.2018.01.009 PMC5994188 29550459

[49] Goncalves OF Soares JM Carvalho S Leite J Ganho A Fernandes-Goncalves A Brain activation of the defensive and appetitive survival systems in obsessive compulsive disorder. Brain Imaging Behav (2015) 9(2):255–63. 10.1007/s11682-014-9303-2 24760279

[50] Cardoner N Harrison BJ Pujol J Soriano-Mas C Hernandez-Ribas R Lopez-Sola M Enhanced brain responsiveness during active emotional face processing in obsessive compulsive disorder. World J Biol Psychiatry (2011) 12(5):349–63. 10.3109/15622975.2011.559268 21781000

[51] van der Knaap LJ van der Ham IJ How does the corpus callosum mediate interhemispheric transfer? A review. Behav Brain Res (2011) 223(1):211–21. 10.1016/j.bbr.2011.04.018 21530590

[52] Johnston JM Vaishnavi SN Smyth MD Zhang D He BJ Zempel JM Loss of resting interhemispheric functional connectivity after complete section of the corpus callosum. J Neuroscience (2008) 28(25):6453–8. 10.1523/JNEUROSCI.0573-08.2008 PMC2738991 18562616

[53] Piras F Piras F Caltagirone C Spalletta G Brain circuitries of obsessive compulsive disorder: a systematic review and meta-analysis of diffusion tensor imaging studies. Neurosci Biobehav Rev (2013) 37(10 Pt 2):2856–77. 10.1016/j.neubiorev.2013.10.008 24177038

[54] Yuan K Qin W Liu P Zhao L Yu D Zhao L Reduced fractional anisotropy of corpus callosum modulates inter-hemispheric resting state functional connectivity in migraine patients without aura. PloS One (2012) 7(9):e45476. 10.1371/journal.pone.0045476 23029036 PMC3454437

[55] Zhou Y Milham M Zuo XN Kelly C Jaggi H Herbert J Functional homotopic changes in multiple sclerosis with resting-state functional MR imaging. AJNR Am J Neuroradiol (2013) 34(6):1180–7. 10.3174/ajnr.A3386 PMC3707620 23348760

[56] Atmaca M Yildirim H Ozdemir H Ozler S Kara B Ozler Z Hippocampus and amygdalar volumes in patients with refractory obsessive–compulsive disorder. Prog Neuropsychopharmacol Biol Psychiatry (2008) 32(5):1283–6. 10.1016/j.pnpbp.2008.04.002 18485556

[57] Chen Y Juhas M Greenshaw AJ Hu Q Meng X Cui H Abnormal resting-state functional connectivity of the left caudate nucleus in obsessive–compulsive disorder. Neuroscience Lett (2016) 623:57–62. 10.1016/j.neulet.2016.04.030 27143323

[58] Xu T Zhao Q Wang P Fan Q Chen J Zhang H Altered resting-state cerebellar–cerebral functional connectivity in obsessive–compulsive disorder. Psychol Med (2019) 49(7):1156–65. 10.1017/S0033291718001915 30058519

[59] Chen Y Meng X Hu Q Cui H Ding Y Kang L Altered resting-state functional organization within the central executive network in obsessive–compulsive disorder. Psychiatry Clin Neurosci (2016) 70(10):448–56. 10.1111/pcn.12419 27377579

